# Recent Progress on the Synthesis of Bipyridine Derivatives

**DOI:** 10.3390/molecules29030576

**Published:** 2024-01-24

**Authors:** Yoshinori Yamanoi

**Affiliations:** Department of Chemistry, School of Science, The University of Tokyo, 7-3-1 Hongo, Bunkyo-ku, Tokyo 113-0033, Japan; yamanoi@chem.s.u-tokyo.ac.jp; Tel.: +81-3-5841-4348; Fax: +81-3-5841-8063

**Keywords:** Suzuki coupling, Stille coupling, Negishi coupling, Ullmann coupling, Wurtz coupling, electrochemical method

## Abstract

Bipyridine and related compounds are starting materials or precursors for a variety of valuable substances such as biologically active molecules, ligands for catalysts, photosensitizers, viologens, and supramolecular architectures. Thus, it is important to classify their synthesis methods and understand their characteristics. Representative examples include methods using homo and heterocoupling of pyridine derivatives in the presence of a catalyst. Because bipyridine compounds strongly coordinate with metal centers, a decrease in catalytic activity and yield is often observed in the reaction system. To address this issue, this review provides insights into advances over the last ~30 years in bipyridine synthesis using metal complexes under both homogeneous and heterogeneous conditions. Moreover, strategies for bipyridine synthesis involving sulfur and phosphorous compounds are examined. These alternative pathways offer promising avenues for overcoming the challenges associated with traditional catalysis methods, providing a more comprehensive understanding of the synthesis landscape.

## 1. Introduction

**Figure 1:**
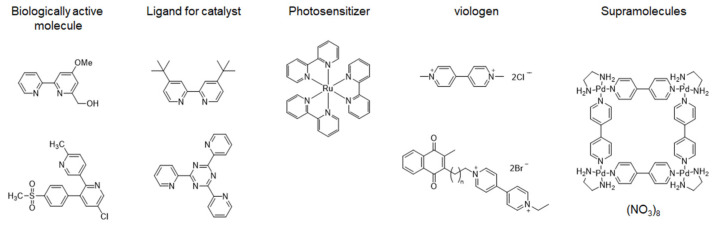
Examples of functional materials containing a bipyridine core.

Bipyridines and their derivatives are extensively used as fundamental components in various applications, including biologically active molecules, ligands in transition-metal catalysis, photosensitizers, viologens, and in supramolecular structures (Figure 1) [1,2,3,4,5,6,7,8,9,10,11]. Many synthetic methods for the preparation of bipyridines have been developed but suffer from low conversion rates and harsh reaction conditions; thus, new methods are now being developed. Problems can be caused by the strong coordination of the product with the metal center decreasing catalytic activity. There are several recent reviews on the synthesis of bipyridine derivatives [12,13] but the aim of this review is to categorize recent research findings over the last ~30 years, focusing on metal-catalyzed cross-coupling reactions (including Suzuki, Negishi, and Stille coupling), metal-catalyzed homocoupling reactions (Ullmann and Wurtz coupling), electrochemical methods, and other innovative techniques. The present review provides a comprehensive overview of recent advances in the synthesis of bipyridines, emphasizing the diverse strategies employed to overcome the challenges associated with conventional methods.

## 2. Metal-Catalyzed Cross-Coupling (Suzuki, Negishi, and Stille Coupling Reactions)

### 2.1. Suzuki Coupling Reaction in Homogeneous Catalytic Systems

**Figure 2:**
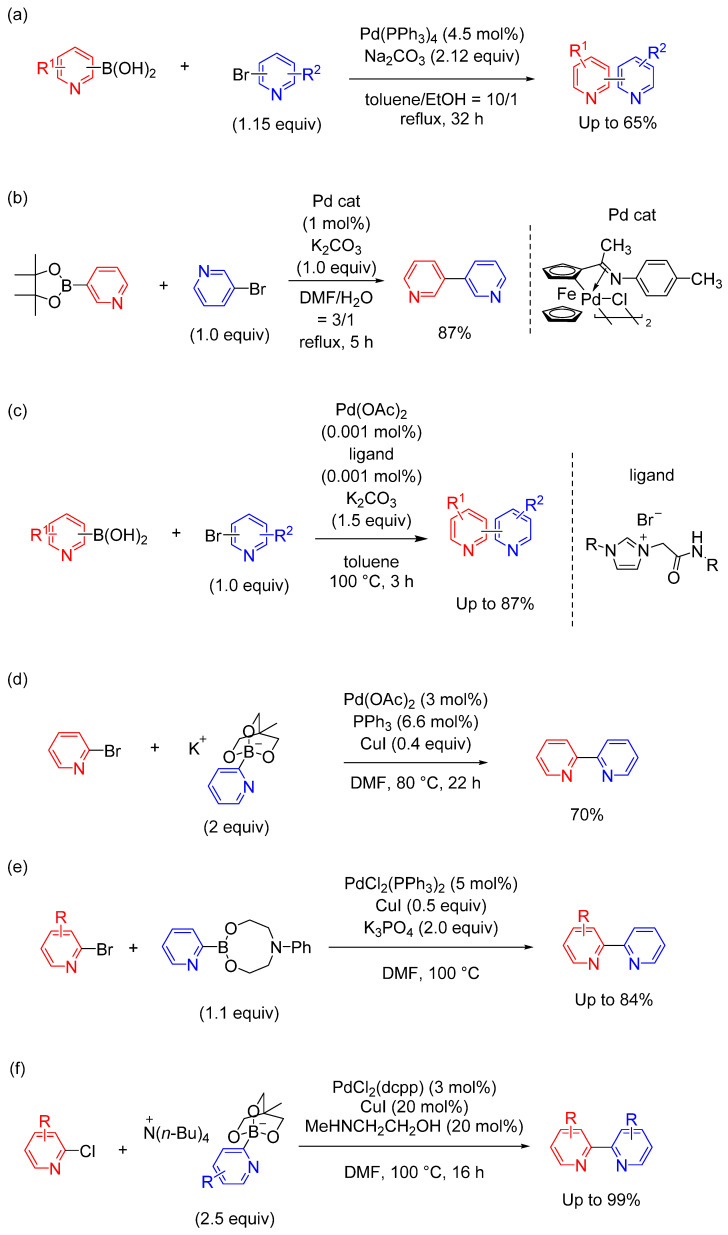
(**a–f**) Representative syntheses of bipyridine-type structures using Suzuki coupling (six examples).

Of the transition-metal cross-coupling reactions, Suzuki coupling is a particularly attractive route for constructing C(sp^2^)–C(sp^2^) bonds and has been widely used for the synthesis of bipyridine structures [14,15,16,17,18,19,20]. However, even though numerous palladium catalysts have been used in Suzuki coupling, a major drawback is the tendency of the bipyridine products to coordinate with the palladium metal, thereby decreasing catalytic activity. The design of the catalytic system is crucial for successful coupling. Several recent representative examples are illustrated in Figure 2.

Matondo et al. and Denton et al. independently reported Suzuki coupling between pyridyl boronic acids and bromopyridines in the presence of Pd(PPh_3_)_4_ and Na_2_CO_3_—typical conditions for Suzuki coupling (Figure 2a) [21,22]. However, the product yields were moderate (50–65%) and a high loading of the Pd catalyst (>10 mol%) was required, possibly due to the aforementioned decrease in catalytic activity during the reaction. To improve the catalytic system, Zhang et al. reported the Suzuki coupling of 3-pyridine boronic pinacol ester with pyridyl halides and a cyclopalladated ferrocenylimine catalyst (Figure 2b) [23]. This palladium catalyst is stable in air and bipyridine derivatives can be synthesized in high yield without the use of inert gas. Kumar et al. reported the synthesis of bipyridines by Suzuki coupling using an imidazolium salt as the ligand for the palladium catalyst (Figure 2c) [24], providing a turnover number of up to 850,000 in the coupling reaction. Thus, boronic acids with 3- and 4-pyridyl groups are stable and can be used to synthesize a variety of compounds via Suzuki coupling. Pharmaceutical bipyridine-based compounds, such as milrinone, were synthesized using these methods. However, the approaches used in these reports were applicable only to 3- or 4-pyridylboronic acid derivatives.

In contrast, 2-pyridyl boronic acid derivatives show poor stability [25], and introducing 2-pyridyl groups into aromatic rings is difficult due to the low efficiency of the catalytic reaction [26]. Therefore, the preparation of stable unsubstituted 2-pyridylboron derivatives remains a synthetic challenge. Yamamoto et al. addressed this challenge by synthesizing bipyridines by coupling [2-PyB(OCH_2_)_3_CHCH_3_]M (M = K^+^, Na^+^, or Li^+^) and 2-bromopyridines (Figure 2d) [27]. Similarly, Jones et al. reported on the Suzuki coupling of 2-pyridineboronic acid *N*-phenyldiethanolamine ester with bromopyridines (Figure 2e) [28]. The boronic ester remains stable during prolonged storage and is commercially available. Couplings were performed with 5 mol% PdCl_2_(PPh_3_)_2_ and 1.1 equivalent of boronic ester relative to the bromopyridine to afford 2,2′-bipyridine-type products in good yield. Sakashita et al. reported that the Suzuki coupling of tetrabutylammonium 2-pyridylborate salts with chloropyridines produced the corresponding bipyridine products with PdCl_2_(dcpp) (dcpp: 1,3-bis(dicyclohexylphosphino)propane) in good-to-excellent yield (Figure 2f) [29] and that the addition of *N*-methyl ethanolamine increased the yield. The reactivity depended on the type of 2-pyridylborate cation. The tetrabutyl ammonium cation had a greater accelerating effect on the reaction, and the order of reactivity was Li^+^ < Na^+^ < K^+^ < Cs^+^ < Bu_4_N^+^. This method enabled the efficient synthesis of many 2,2′-bipyridines. The addition of CuI as a co-catalyst helped increase the yield of 2,2′-bipyridine. The exact role of CuI in this reaction is unknown, but a similar effect of copper salts has previously been used in analogous coupling reactions of heteroarylboron compounds, with the addition of CuI improving product yield.

### 2.2. Negishi and Stille Coupling Reactions in Homogeneous Catalytic Systems

**Figure 3:**
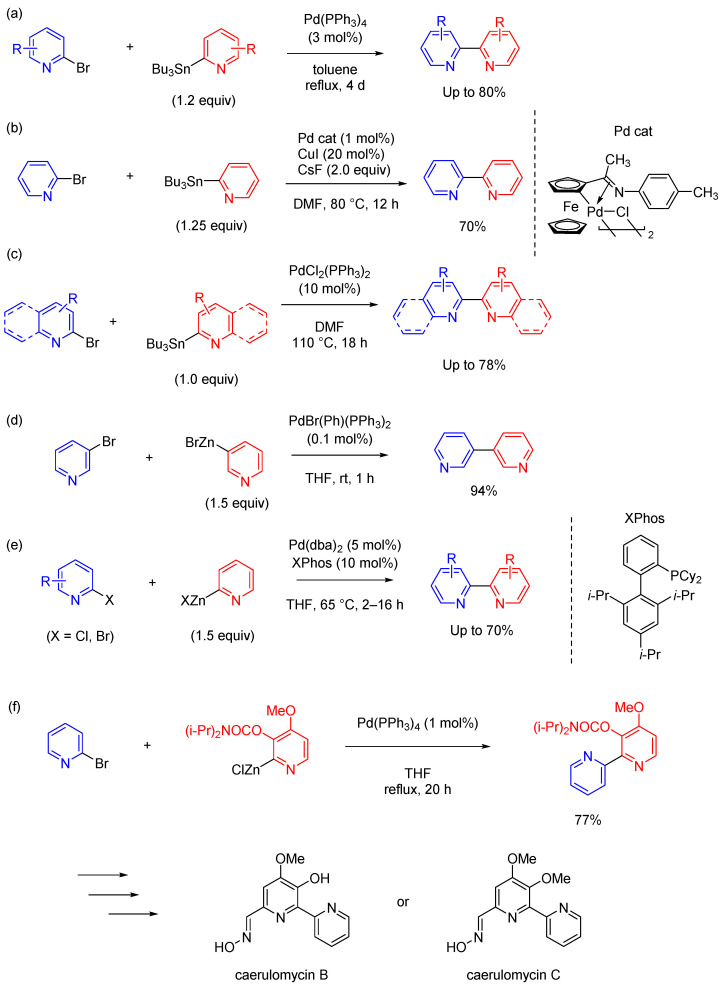
Synthesis of bipyridine (six examples). (**a**–**c**) Stille coupling. (**d**–**f**) Negishi coupling.

Stille coupling is a synthetic method that uses organotin compounds [30,31] and is highly reactive, such that it can proceed even in systems not amenable to Suzuki coupling [32]. However, a notable drawback of Stille coupling is the toxicity and hazardous nature of organostannyl compounds. Heller and Schubert reported the use of Stille-type cross-coupling to prepare various 2,2′-bipyridines (Figure 3a) [33]. Terpyridine derivatives can be synthesized using this technique using chelating ligands. Ma et al. reported Stille coupling between 3- or 2-stannylpyridines and bromopyridines catalyzed by 1 mol% cyclopalladated ferrocenylimine with tricyclohexylphosphine in the presence of CuI (additive) and CsF (base) (Figure 3b) [34]. Verniest et al., presented the synthesis of bipyridines through the Stille reaction between stannylated pyridines and bromopyridines in the presence of PdCl_2_(PPh_3_)_2_ (Figure 3c) [35]. Biquinolinyl compounds can be synthesized by using 2-quinolinyl stannanes and 2-bromo-substituted quinolines using the same reaction conditions. This approach can be extended to the synthesis of lavendamycin and analogues starting from functionalized quinolines and β-carboline building blocks. Although Stille coupling is useful in the synthesis of bipyridine derivatives, the organotin molecules used as reactants are highly toxic.

In related research, bipyridines were synthesized using Negishi coupling as an alternative synthetic method. Tang et al. reported particularly good results using PdBr(Ph)(PPh_3_)_2_ as a catalyst (Figure 3d) [36]. The catalyst, termed a post-oxidative addition precatalyst, can be easily prepared and is highly stable in air and in the presence of moisture. The catalyst showed high activity in Negishi coupling [37]. Luzung et al., and Downs et al., reported the preparation of 2,2′-bipyridine derivatives by Negishi coupling between 2-pyridyl zinc halides and bromopyridines in the presence of Pd(dba)_2_ and XPhos [38,39]. This method complements current reactions for the coupling of 2-pyridyl organometallic reagents and will likely aid drug discovery and development. Mongin et al. reported Negishi coupling between (3-(diisopropylcarbamoyl)-4-methoxypyridin-2-yl)zinc (II) chloride and 2-bromopyridine in the presence of a catalytic amount of Pd(PPh_3_)_4_ (Figure 3f) [40]. The reaction proceeded in THF under reflux for 20 h. The coupling product can be converted into caerulomycin B or caerulomycin C, which are useful as STAT1-signaling inhibitors and immunosuppressants, showing their application in medicinal chemistry.

### 2.3. Other Cross-Coupling Reactions in Homogeneous Catalytic Systems

**Figure 4:**
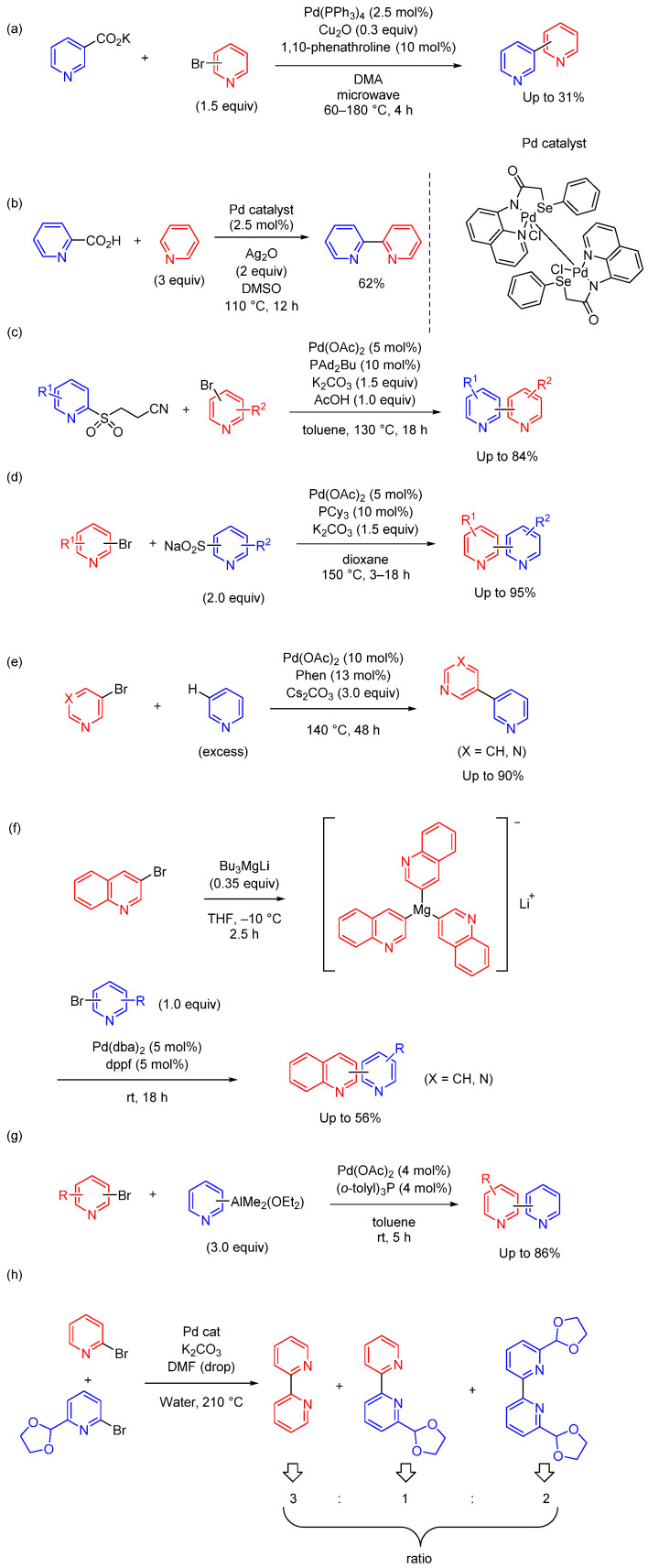
Synthesis of bipyridine derivatives using other cross-coupling reactions in homogeneous catalytic systems (eight examples). (**a**,**b**) Decarboxylative cross-coupling. (**c**,**d**) Desulfonylative cross-coupling. (**e**) Pd-catalyzed C-3 selective arylation of pyridine. (**f**) Pd-catalyzed coupling of lithium tri(3-quinolinyl)magnesite and bromopyridine. (**g**) Pd-catalyzed coupling of pyridyldimethylaluminums with bromopyridines. (**h**) Pd-catalyzed homocoupling and heterocoupling of 2-bromopyridines.

This section discusses the synthesis of bipyridines using metal catalysts which cannot be classified as Suzuki, Stille, or Negishi coupling. The synthesis of biaryl and heterobiaryl compounds via decarboxylative cross-coupling of aromatic carboxylates has been demonstrated [41,42]. Using this method, Chennamanneni et al. reported the microwave-assisted Pd-catalyzed decarboxylative cross-coupling of pyridyl carboxylates with bromopyridines (Figure 4a) [43]. 1,10-Phenathroline improved the yield, showing that the bite angle of the bidentate *N*,*N*-ligand is critical in the decarboxylation process. They achieved the decarboxylative cross-coupling of both 3-pyridyl and 4-pyridyl carboxylates with aryl bromides, allowing the synthesis of 3- or 4-arylpyridines of interest to pharmacological applications.

Expanding on this approach, Singh et al. developed dinuclear palladium pincer complexes incorporating a Pd–Pd bond based on *N*,*N*,Se-ligands [44]. Bipyridine compounds can be synthesized in good yield by combining the decarboxylation of picolinic acid and the C–H activation of pyridine using this complex as a catalyst (Figure 4b). In this reaction, Ag_2_O was used as an oxidant, resulting in the formation of Ag(I)–pyridine. 2,2′:6′,2″-Terpyridine can be synthesized by using 2,6-pyridinecarboxylic acid in place of picolinic acid. This study not only introduced an efficient route to bipyridine synthesis but also demonstrated the adaptability of this method by using different starting materials and different synthetic applications.

Cook et al. reported the synthesis of bipyridine derivatives by the coupling of pyridine sulfinate with bromopyridines (Figure 4c) [45]. β-Nitrile pyridylsulfones were used as efficient base-labile latent sulfinate reagents in the Pd-catalyzed cross-coupling reaction. No acrylonitrile byproduct was observed and was presumably removed by evaporation. The scope of electrophilic partners is broad, displaying good tolerance of multiple functional groups and substitution patterns, delivering the desired cross-coupled products in good-to-high yield. The method allowed access to a diverse range of 2-arylpyridines, demonstrating its utility for the synthesis of pharmaceutical molecules. In a related study, Markovic et al. reported that 2-pyridyl sulfinate salts (sodium or lithium) are effective coupling partners in the Pd-catalyzed cross-coupling reaction with bromopyridines (Figure 4d) [46]. The scope of halide partners is wide and allows the preparation of a broad range of bipyridine derivatives. They applied this method to medicinally relevant molecules, including in library synthesis. These two studies not only expanded the scope of bipyridine synthesis but also highlighted the versatility and practicality of these methods in medicinal chemistry.

Transition-metal-catalyzed C–H arylation of heteroarenes is another recent promising strategy. Ye et al. reported the synthesis of dipyridines through the Pd-catalyzed non-directed C-3 arylation of pyridine (Figure 4e) [47] and synthesized 3,3′-bipyridine and 5-(pyridine-3-yl)pyrimidine in good yield. The utility of this method has been demonstrated in the concise synthesis of pyridine-based drugs. Dumouchel et al. reported Pd-catalyzed cross-coupling between bromopyridines and lithium tri(3-quinolinyl)magnesite, prepared by the bromine–magnesium exchange of 3-bromoquinoline and Bu_3_MgLi [48]. This method provided functionalized quinoline derivatives in moderate yield (Figure 4e). Chen et al., reported the cross-coupling of pyridyl aluminum reagents with pyridyl bromides using Pd(OAc)_2_ and (*o*-tolyl)_3_P. The reactions proceeded efficiently at room temperature without a base. Although the scope of this approach is limited to unsubstituted pyridyl aluminum reagents, the product yields were good [49]. Nicasio-Collazo et al, reported the homo and heterocoupling of bromopyridines by neophylpalladacycle (Figure 4g) [50]. The reaction between 2-BrPy and 2-Br-6-(C_3_H_5_O_2_)-Py gave a mixture of 2,2′-bipyridine, 6-(1,3-dioxolan-2-yl)-2,2′-bipyridine, and 6,6′-di(1,3-dioxolan-2-yl)-2,2′-bipyridine in a ratio of 3:1:2, as determined by ^1^H NMR measurements.

### 2.4. Cross-Coupling Reactions in Heterogeneous Catalytic Systems

**Figure 5:**
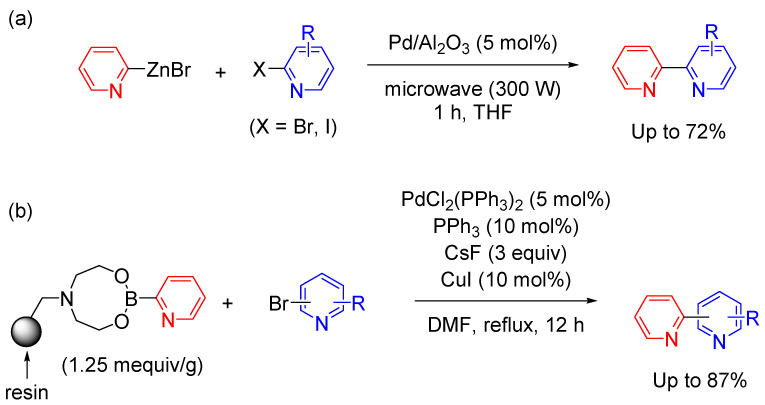
Cross-coupling reactions in heterogeneous catalytic systems (two examples). (**a**) Negishi coupling with Pd/Al_2_O_3_ under microwave irradiation. (**b**) Suzuki coupling with polystyrene-supported 2-pyridylboron.

Heterogeneous catalytic systems have major advantages, including easy production and purification, and amenability to reuse. Representative examples are shown in Figure 5. Vici et al. reported that Ni/Al_2_O_3_–SiO_2_ (50 mol%) or Pd/Al_2_O_3_ (5 mol%) afforded 2,2′-bipyridine products through Negishi coupling between 2-pyridyl zinc bromide and 2-bromopyridine derivatives (Figure 5a) [51]. The yields were dramatically enhanced by microwave irradiation (300 W), with the reaction complete within 1 h. No coupling product was obtained when pyridyl magnesium bromides were used instead of pyridyl zinc reagents.

Several nanoparticles have been reported to have highly catalytic surfaces and high turnover numbers. Palladium nanoparticles are the best-studied examples of these catalysts. The surface of nanoparticle catalysts must be protected with a protective agent, and polymers, dendrimers, surfactants, and organic ligands have been studied as catalyst surface stabilizers. Gros et al. reported an example in which a carrier can be easily recovered and reused for the Suzuki coupling of polystyrene-supported 2-pyridylboron with bromopyridines, providing bipyridines in high yield (Figure 5b) [52]. The polymer-supported material is a stable source of 2-pyridylboranate. This study provided new perspectives for Suzuki coupling in combinatorial chemistry for preparing bipyridine derivatives. The ease of recovery and reusability of the supported catalysts makes this approach practical and is consistent with the growing interest in sustainable synthesis methods.

## 3. Metal-Catalyzed Homocoupling Reactions (Wurtz Coupling and Ullmann Coupling)

**Figure 6:**
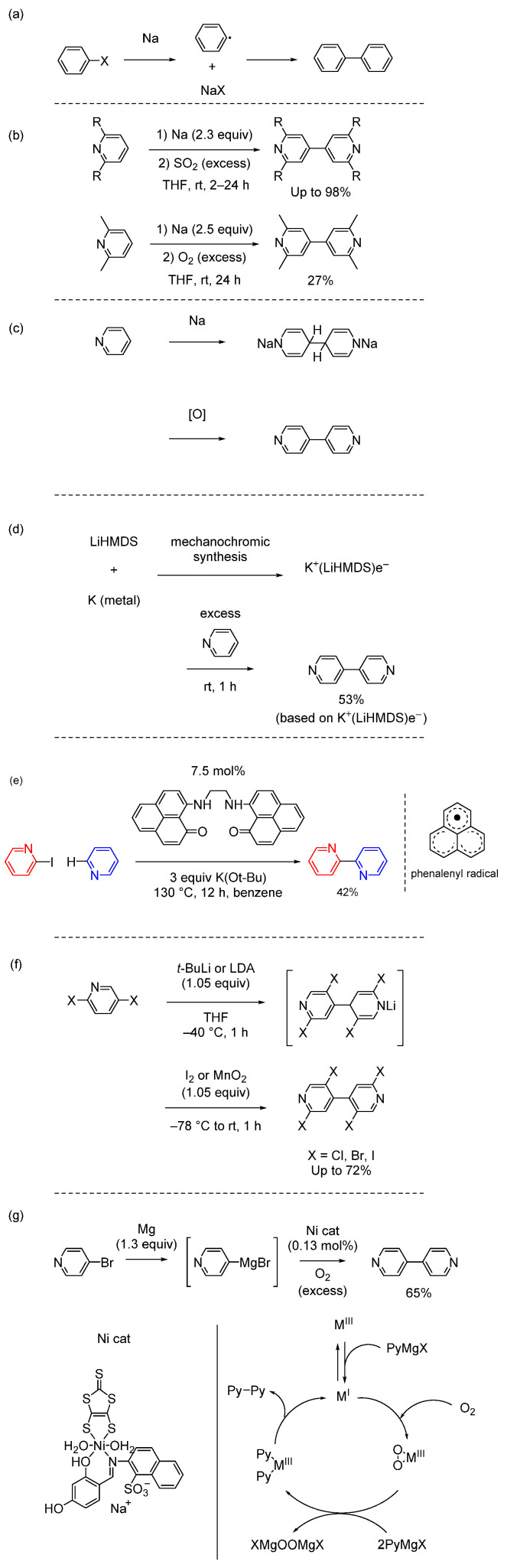
(**a**) Reaction mechanism of the Wurtz coupling of aryl halides using a Na dispersion. (**b**) Wurtz coupling of pyridine derivatives using a Na dispersion and oxidant (two examples). (**c**) Reaction mechanism of the Wurtz coupling of pyridines. (**d**) Synthesis of 4,4′-bipyridine with the stable electride reagent K^+^(LiHMDS)e^−^. (**e**) C–H functionalization employing a bis-phenalenyl compound and K(Ot-Bu). (**f**) Synthesis of 4,4′-bipyridine derivatives via dimerization. (**g**) Proposed mechanism for the metal-catalyzed homocoupling reaction of Grignard reagents in the presence of oxygen.

The Wurtz reaction is useful for obtaining symmetrical bipyridines [53]. Wurtz coupling typically involves reacting organic halides with a Na dispersion (Figure 6a). Bipyridines can be synthesized by reacting pyridines with the Na dispersion and then reacting with an excess of oxidizing agent (Figure 6b) [54,55]. The reaction mechanism is shown in Figure 6c. This method provides a valuable tool for accessing diverse bipyridine derivatives.

The single electron transfer (SET) approach requires Na metal, which has limited its utility in bipyridine synthesis. Davison et al. reported the facile synthesis of the room temperature-stable electride reagent K^+^(LiHMDS)e^−^ (HMDS: 1,1,1,3,3,3-hexamethyldisilazide) from K metal and LiHMDS via mechanochemical ball milling at a 20 mmol scale. The reagent is versatile in mediating transition-metal-free pyridine C–H activation and C–C coupling (Figure 6d) [56].

In related research, Banik et al. reported the synthesis of bipyridines through a transition-metal-free C–H functionalization employing a bis-phenalenyl compound and K(O*t*-Bu) (Figure 6e) [57]. The reaction mechanism involves a SET from a phenalenyl radical to generate a reactive pyridyl radical from the halogenated pyridine, which forms a C(sp^2^)–C(sp^2^) bond with pyridine through a SET. The presence of organic radicals was confirmed by electron spin resonance measurements. Because the yield of the biheteroaryl compound is moderate using this method, new methods providing improved yields are desirable.

As an application of Wurtz coupling, Abboud et al. synthesized polyhalogenated 4,4′-bipyridines by coupling 4-lithiodihalopyridines with an oxidizing agent (I_2_ or MnO_2_) (Figure 6f) [58]. The reaction mechanism was studied by isolating and characterizing several byproducts. The drawbacks of this method are that the bipyridine derivative cannot be synthesized unless there are multiple halogen substituents in the pyridine ring and the product is generated in moderate yield.

An improved method for the Wurtz coupling approach is the transition-metal-catalyzed homocoupling of Grignard reagents, which is one of the most efficient synthetic methods for constructing symmetrical bipyridyl backbones [59]. Many reports have been published of coupling reactions using metal reagents, but these methods use a two-step synthetic route in which organometallic compounds are initially prepared and isolated, followed by their subsequent conversion into bipyridine products in the presence of an oxidant as a separate reaction. The demand for stoichiometric amounts of organic oxidant limits the utility of this approach for large-scale syntheses. Bhat and Bhat reported a metal-catalyzed procedure for the homocoupling of Grignard reagent prepared in situ to give symmetrical bipyridines in a single step [60]. A low-valent metal species is generated in the presence of Grignard reagent in situ [61]. The reaction is performed in the presence of oxygen as an oxidant and the reaction mechanism is shown in Figure 6g. The absorption spectrum of the reaction solution showed a peak derived from a peroxo-M(III) species at 420 nm. This chemical species is likely the key chemical species in the transformation. Importantly, the reaction system demonstrates notable tolerance towards chloro-, nitro-, cyano-, and hetero-aryl functionalities, resulting in good-to-high yields of symmetrical biaryls. Moreover, this process requires a minimal amount of catalyst, enhancing its efficiency.

**Figure 7:**
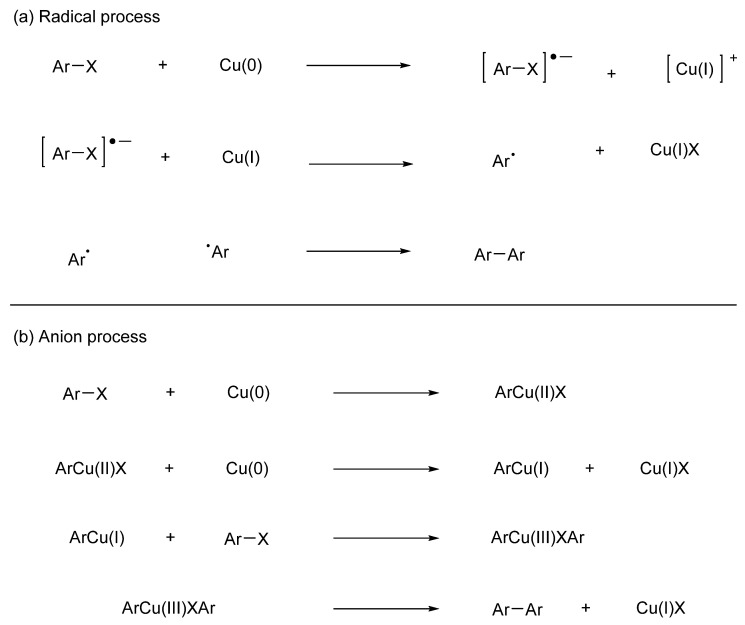
Reaction mechanism of Ullmann coupling using copper metal. (**a**) Radical process. (**b**) Anion process.

Ullmann coupling is a valuable technique for obtaining symmetrical bipyridines [62,63]. The original and convenient route to synthesizing symmetric bipyridines is the stoichiometric copper-mediated homocoupling of aryl halides [64,65,66,67,68,69]. Figure 7 shows the reaction mechanism when copper metal is used as a typical example. A radical reaction mechanism and an anionic reaction mechanism have been considered, although it remains unknown which mechanism occurs. The use of high temperatures (>200 °C), poor substrate scope, and need to use stoichiometric amounts of copper reagent has limited the utility of these reactions. Nonetheless, Ullmann coupling remains an important method for obtaining symmetrical bipyridines. Advances in reaction conditions and exploration of alternative methods may improve the practicality and efficiency of this synthetic route.

**Figure 8:**
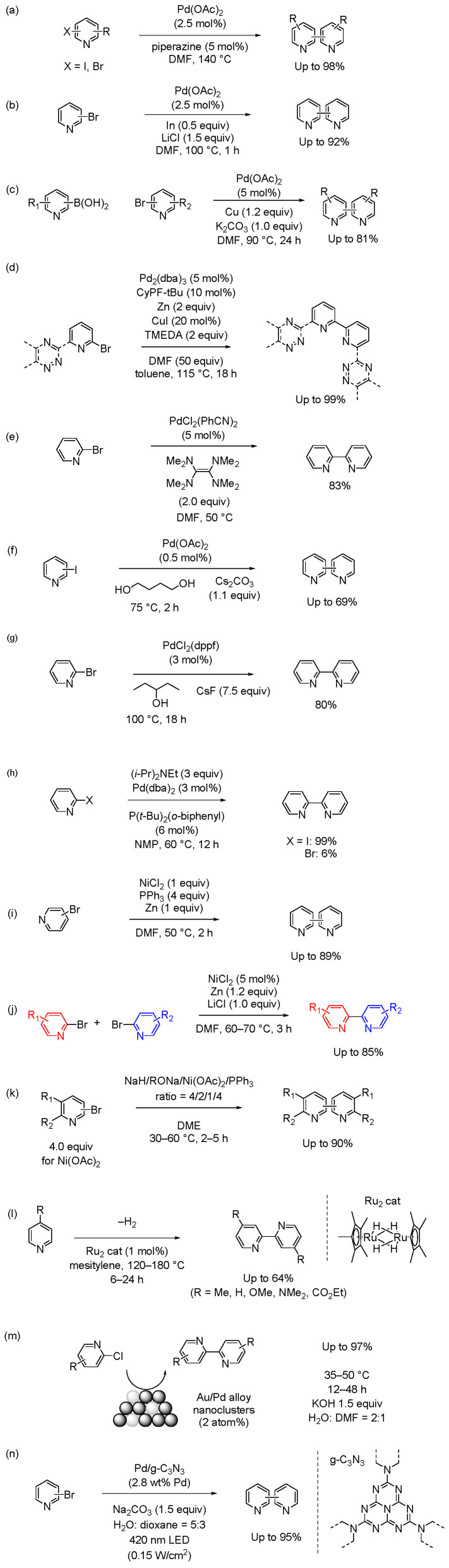
Representative examples of the homocoupling of pyridine derivatives in the presence of catalysts (14 examples). (**a**–**d**) Pd-catalyzed homocoupling of halopyridines. (**e**) Reductive coupling of bromopyridines with PdCl_2_(PhCN)_2_/TDAE. (**f**,**g**) Pd-catalyzed homocoupling of iodopyridines in alcoholic solvents. (**h**) Pd-catalyzed homocoupling without reducing agents. (**i**,**j**) Ni-catalyzed homocoupling of bromopyridine. (**k**) Homocoupling of pyridyl halides with NaH/RONa/Ni(OAc)_2_/PPh_3_. (**l**) Homocoupling of pyridines with Ru complex. (**m**) Homocoupling of chloropyridines using Au/Pd alloy nanoparticle. (**n**) Homocoupling of bromopyridines using Pd/g-C_3_N_3_.

Bipyridine compounds can be synthesized in good yield by performing two oxidative additions of halogenated pyridine in the presence of a palladium catalyst and a reducing agent. Recent representative examples are shown in Figure 8. For example, the combination of Pd(OAc)_2_ and piperazine in DMF at 140 °C facilitates the homocoupling of bromopyridines (Figure 8a) [70]. Although the reaction requires a high temperature (140 °C), it is operationally straightforward and exhibits good substrate compatibility.

Lee reported that the treatment of bromopyridines in the presence of Pd(OAc)_2_ with indium and LiCl efficiently produced bipyridines through homocoupling in good-to-excellent yield (Figure 8b) [71]. Although the mechanism of the coupling reactions based on bimetallic systems has not been established, the key step of the transformation proceeded via a direct transfer from indium to palladium (II) species. Li et al. reported bimetallic Ullmann coupling of bromopyridines in the presence of stoichiometric copper powder and a catalytic amount of Pd(OAc)_2_ (Figure 8c). The catalytic system showed good tolerance to different functional groups in good yield under relatively mild conditions [72]. The coupling process was promoted via radicals generated by redox interactions between Cu(0) and Pd(IV) species in the heated system. The results indicated the robust tolerance of this method for bromopyridines with different functional groups and various symmetric bipyridines were efficiently prepared with good chemical yields. Carrick and Waters reported the synthesis of 2,2′-bipyridines and bis-1,2,4-triazinyl-2,2′-bipyridines via a Pd-catalyzed Ullmann-type reaction in the presence of Zn, Cu(I), and TMEDA (Figure 8d) [73], probably via a synergistic transformation dependent on the cooperativity of Pd(II), Zn(0), and Cu(I). The prepared bipyridine derivatives were examined in separation experiments on spent nuclear fuel, emphasizing the practical applications of this synthetic method. These processes highlight the unique reactivity achieved by using bimetallic systems and provide new avenues for bipyridine synthesis.

Kuroboshi et al. reported that the PdCl_2_(PhCN)_2_-promoted reductive coupling of bromopyridines proceeded smoothly to afford the corresponding bipyridines in the presence of tetrakis(dimethylamino)ethylene (TDAE) as an organic reductant in good yield (Figure 8e) [74]. TDAE is a mild reductant and hardly reduces functional groups. The reductive coupling reaction was initiated by the reduction of Pd(II) with TDAE, generating Pd(0). Although the homocoupling of 2-bromopyridine and 4-bromopyridine occurred in this reaction, no reaction occurred with 3-bromopyridine.

Several research groups have investigated reaction systems using alcohol as both a solvent and reducing agent. For example, Huang et al. reported the synthesis of bipyridine via Pd-catalyzed reductive homocoupling in 1,4-butanediol in air (Figure 8f) [75]. The reaction proceeded in the presence of 0.01 mol% Pd(OAc)_2_ as a catalyst, and 1,4-butanediol was used as the *O*,*O*-ligand, solvent, and reductant, so no extra reducing agents and ligand were required in the catalytic system. This method benefits from low Pd catalyst loading and mild reaction conditions. Zeng et al. reported that Pd(dppf) catalyzed the reductive homocoupling of bromopyridine or iodopyridine in 3-pentanol. X-ray photoelectron spectroscopy indicated that the oxidation of 3-pentanol is involved in the in situ regeneration of the reductive Pd^0^(dppf) active species; 3-pentanol functions as a reducing agent, and is converted to 3-pentanone. This catalytic system is simple, and the elimination of additives simplifies product separation and purification (Figure 8g) [76].

Examples of Pd-catalyzed homocoupling of halopyridines were recently reported without a reducing agent [77,78,79]. For example, Manoso and DeShong demonstrated various coupling reactions using Pd catalysts with high catalytic activity (Figure 8h) [80]. 2-Iodopyridine was converted to 2,2′-dipyridyl in good yield in the presence of Pd(dba)_2_, P(*t*-Bu)_2_(*o*-biphenyl), and (*i*-Pr)_2_NEt. However, employing the same conditions with 2-bromopyridine provided only trace amounts of the coupled product.

This reaction proceeds even with a cost-effective nickel catalyst. Traditionally, reductive coupling with stoichiometric amounts of hydrated NiCl_2_, PPh_3_, and Zn affords bipyridines in good yield (Figure 8i) [81], although using this catalytic method for the synthesis of bipyridines from halopyridines led to low bipyridine yields due to the competing reductive dehalogenation of the substrates. Liao et al. reported a facile synthetic approach for symmetrical and unsymmetrical 2,2′-bipyridines through Ni-catalyzed reductive couplings of 2-halopyridines (Figure 8j) [82]. The couplings were efficiently catalyzed by NiCl_2_·6H_2_O without external ligands to give 2,2′-bipyridines in high yield. 3,3′-Bipyridines were not synthesized by the catalytic systems, suggesting that the product, 2,2′-bipyridine derivatives, acted as ligands for nickel (II), facilitating the smooth progress of the coupling reaction. Various 2,2′-bipyridines were synthesized.

Li et al. reported that 2,2′,6,6′-tetramethyl-4,4′-bipyridine was obtained in high yield by the homocoupling of 4-bromo-2,6-dimethylpyridine under mild conditions with NiBr_2_(PPh_3_)_2_, Et_4_NI, and zinc powder [83]. They investigated the electrochemical properties of the viologen derivatives at the 2,2′,6,6′-positions of the 4,4′-bipyridine core rings. Many coordinating pyridine derivatives were synthesized. The examples provided suggest that diverse complexants can be obtained for structure–activity relationship studies in separation systems.

Vanderesse et al., reported effective Ullmann coupling of pyridyl halides using the Ni catalyst prepared from NaH/*t*-BuONa/Ni(OAc)_2_/PPh_3_ (Figure 8k) [84]. The optimal component ratio was determined to be 4:2:1:4, and DME and *t*-BuONa were the best solvent and activating alkoxides, respectively. The side reaction that gave the reduced product was suppressed to about 20%.

Dehydrogenative coupling of functionalized pyridines with direct C–H bond activation is a promising economical and environmentally friendly route. Kawashima et al. reported that the diruthenium tetrahydrido complex, Cp*Ru(μ-H)_4_RuCp*, catalyzed the dimerization of 4-substituted pyridines [85]. The reaction proceeded through the cleavage of C–H bonds with the Ru complex (Figure 8l), and the 2-position of the pyridines was the reaction site, producing the corresponding bipyridine derivatives. The reactivity of the dehydrogenative coupling depended on the substituent on the pyridine ring, and a higher pKa provided the product in good yield. No byproducts, such as terpyridine, were formed in the transformation. Bis(µ-pyridyl) and µ-η^2^:η^2^-bipyridine-coordinated Ru complexes were isolated as intermediates in the catalytic cycles.

Several Ullmann couplings using heterogeneous catalysts have been reported. Dhital et al. reported bimetallic gold–palladium alloy nanoclusters as an effective catalyst for the Ullmann coupling of chloropyridines under ambient conditions (Figure 8m) [86]. The Ullmann coupling product was not observed when monometallic Au and/or Pd clusters were used as catalysts. In contrast to conventional transition metal catalysts, 2-chloropyridine exhibited higher reactivity than 2-bromopyridine. UV-vis and inductively coupled plasma–atomic emission spectroscopy measurements revealed that a large amount of Pd(II) leached during the coupling with 2-bromopyridine compared with that with 2-chloropyridine, suggesting that leaching may be important in decreasing reactivity. Tian et al. reported that the light-induced oxidative half-reaction of water splitting could be coupled with the reduction of bromopyridines (Figure 8n) [87]. This strategy enabled various aryl bromides to undergo reductive coupling with Pd/graphite phase carbon nitride as the photocatalyst, providing a pollutant-free method for synthesizing bipyridine skeletons. Additionally, the use of green visible light had further advantages, including mild conditions and good functional group tolerance. Despite some drawbacks, such as the need for environmentally unfriendly dioxane and the addition of Na_2_CO_3_, this method allows water to be used as a reducing agent, which may lead to the development of cleaner procedures for various organic reactions. The use of photocatalytic water splitting is particularly desirable in green chemistry.

## 4. Electrochemical Methods

**Figure 9:**
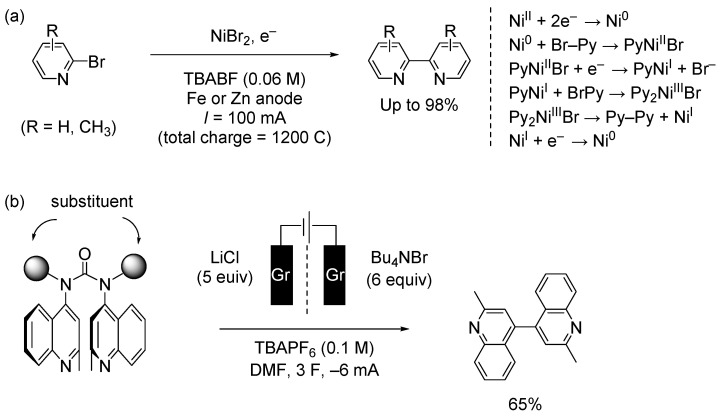
Electrochemical methods (two examples). (**a**) Coupling of 2-bromopyridines. DMF, TBABF, Fe or Zn anode, room temperature (left). Reaction mechanism (right). (**b**) Electrochemical intramolecular coupling reaction of pyridyl urea derivatives. Gr: graphite electrode.

Electrochemical methods are among the most promising synthetic approaches from an environmental perspective, avoiding toxicity and high cost, especially for the synthesis of pharmaceutical molecules. However, few effective methods of bipyridine synthesis have been reported [88]. Navarro and colleagues reported the nickel-catalyzed electroreductive homocoupling of bromopyridines in an undivided cell using a Zn or Fe anode. By optimizing the reaction conditions, they efficiently synthesized heterocoupled products (Figure 9a left) [89,90]. The method is simple and efficient, with an isolated yield of up to 98% using DMF as the solvent. They also explored heterocoupling reactions, and statistical yields were observed for the heterocoupling. For example, a reasonable isolated product yield of 6,6″-dimethyl-2,2′:6′,2″-terpyridine was observed in the reaction between 2,6-dichloropyridine and 2-bromo-6-methylpyridine. The catalytic cycle is shown in Figure 9a (right). The advantages of this method are that no PPh_3_ ligand is required, and that active Ni(0) is generated using a constant current density. However, it is not controlled by a complete electrode reaction.

Stammers et al. reported an electrochemical approach to the synthesis of bipyridine derivatives from *N*,*N*′-dipyridylureas (Figure 9b) [91]. Urea and a graphite electrode were added to the cathodic H-cell, whereas a graphite electrode was added to the anodic H-cell. DMF was used as a solvent. The reaction was subjected to electrolysis at 3 F mol^−1^ and −6 mA under a N_2_ atmosphere. The electrochemical transformation was important as an intermolecular reaction. Sterically hindered substrates could also be used for the synthesis of bipyridine derivatives. Conformational alignment of the arenes in the *N*,*N*′-diaryl urea intermediates promoted C–C bond formation following single-electron reduction. This method supports complementary reactivity to most metal-catalyzed coupling reactions. The procedure is operationally simple and represents an improvement over other synthesis of disubstituted bipyridines.

## 5. Other Methods

In this section, synthetic methods that cannot be classified into the other categories in this review are discussed. The development of alternative methods, such as transition-metal-free systems, for cross-coupling remains desirable. To prepare bipyridine derivatives, the use of nonmetallic third period elements, such as sulfur and phosphorus, has been considered because of their high maximum coordination numbers.

**Figure 10:**
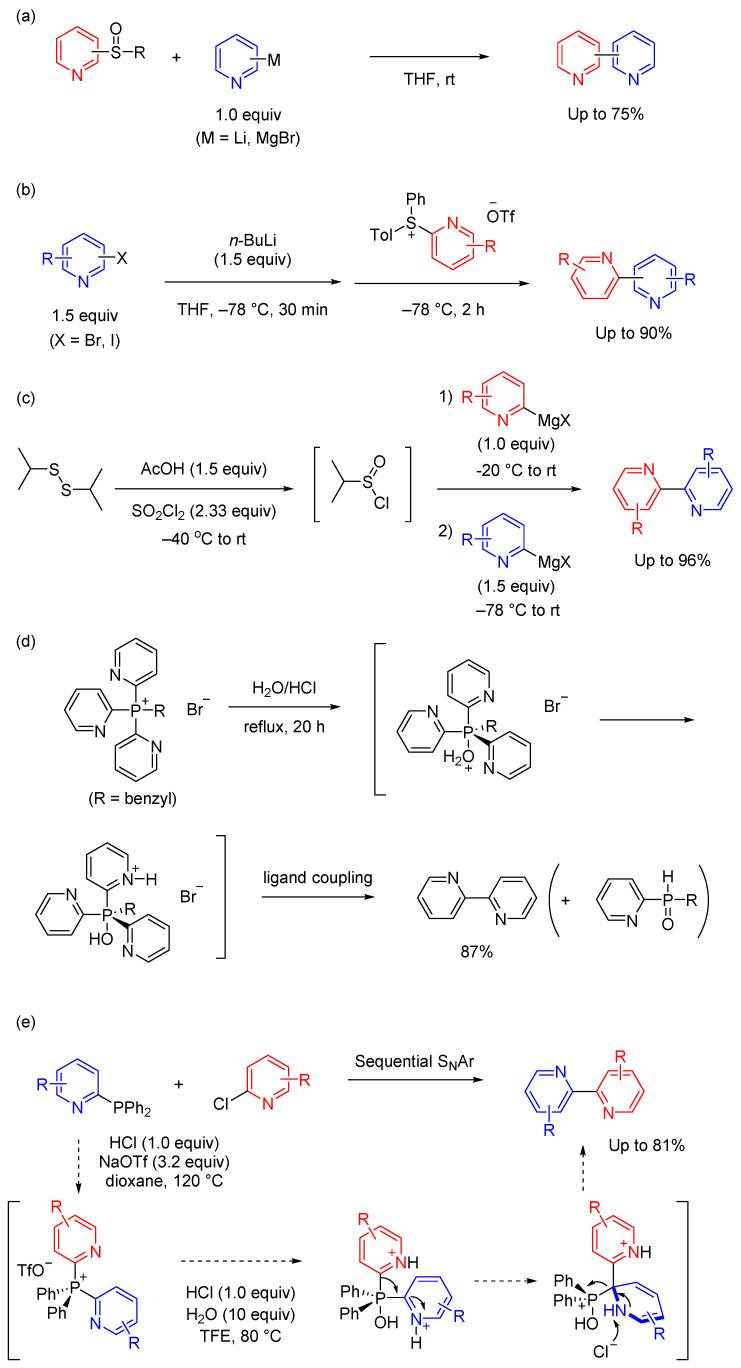
Other methods (five examples). (**a**–**c**) Sulfur-mediated synthesis of bipyridine derivatives. (**d**,**e**) Phosphorous-mediated synthesis of bipyridine derivatives.

Sulfur-mediated organic synthesis is thus a rich area of study for C–C bond formation [92,93]. For example, Furukawa and colleagues demonstrated that the addition of pyridyl lithium (or pyridyl magnesium bromide) to pyridyl aryl sulfoxides led to the formation of bipyridine derivatives via sulfurane intermediates (Figure 10a) [94,95]. The mechanism for the formation of bipyridyls has been proposed as an initial attack of the Grignard reagent on the sulfinyl sulfur atom to afford the sulfurane as an intermediate, from which the two pyridyl groups couple selectively participates while the phenyl (or tolyl) group on the sulfoxide does not participate. This selective C–C bond formation makes the mechanism precise. Several sulfur-mediated bipyridine syntheses have been reported that improve on this approach [96,97,98].

Duong et al. described the synthesis of pyridylsulfonium salts and their application to the preparation of bipyridine derivatives through a ligand coupling reaction [99]. The key intermediate sulfonium salts were obtained by Cu(OTf)_2_-catalyzed S-selective arylation of *p*-tolylpyridyl sulfide with Ph_2_IOTf. To demonstrate the synthetic utility of this approach, the resulting pyridylsulfonium salts were used in a scalable transition-metal-free coupling protocol, yielding functionalized bipyridines with remarkable functional group tolerance (Figure 10b). This modular methodology permitted selective introduction of functional groups from commercially available pyridyl halides, facilitating the synthesis of both symmetrical and unsymmetrical 2,2′- and 2,3′-bipyridines. Importantly, the bipyridine was formed via a sulfuran intermediate, highlighting the utility of this method in obtaining structurally diverse bipyridine compounds. Zhou et al. reported a sulfinyl(IV) chloride-mediated cross-coupling involving two pyridyl Grignard reagents (Figure 10c) [100]. The intermediate in this transformation, isopropyl sulfinyl(IV) chloride, was readily obtained from diisopropyl disulfide. The addition of successive pyridyl nucleophiles to sulfinyl (IV) chloride facilitated the formation of a trigonal bipyramidal sulfurane intermediate. The subsequent reductive elimination afforded the bispyridyl products in a practical and efficient manner. Many functional groups are tolerated under the reaction conditions, allowing rapid access to molecular complexity. In contrast to transition metal-catalyzed couplings, this reaction is uniquely suited to the preparation of Lewis base substrates, which are difficult to couple under classical conditions.

Phosphorus-mediated C–C bond formation has attracted much attention. The synthesis of bipyridines via a phosphorus-ligand coupling reaction has been investigated and the feasibility of this method has been demonstrated with a variety of precursors [101,102]. Uchida et al. revealed that treating tri(2-pyridyl)benzyl phosphonium bromide with acidic water provided 2,2′-bipyridine in good yield (Figure 10d) [103] with no formation of 2-benzylpyridine, suggesting that the benzyl group could not approach the axial position of the intermediate in aqueous conditions. Consequently, this method is excellent for synthesizing symmetrical bipyridine compounds. Inspired by this method, Boyle et al. reported the formation of bipyridines by coupling pyridylphosphines with chloropyridines. The reaction proceeded via a tandem S_N_Ar-ligand-coupling sequence (Figure 10e) [104] via heating phosphine and chloropyridine in dioxane with HCl and NaOTf to form the bis-heterocyclic phosphonium salt, followed by further addition of HCl and H_2_O in trifluoroethanol, allowing the ligand coupling reaction to proceed. A diverse set of bis-azine biaryl products were formed in good-to-excellent yield, including substitution patterns such as 2,2′-bipyridines that are challenging for traditional metal-catalyzed approaches. The abundance of chloroazines, simple protocols, and valuable bispyridine products make this approach useful for medicinal chemists.

## 6. Summary

This review provides a comprehensive overview of recent advances in the synthesis of bipyridine derivatives. The synthetic methods are categorized as metal-catalyzed heterocoupling, Ullmann coupling and Wurtz coupling, electrochemical approaches, and other methods. The development of efficient synthetic methods for bipyridine derivatives is promising for the synthesis of diverse functional materials. Each synthetic approach highlighted in this review has unique characteristics, and efforts are continuing toward synthetic methods that are suitable for the entire range of bipyridine derivatives. Future challenges lie in implementing these methods on an industrial scale to ensure efficient synthesis. Continuous exploration and optimization of these methodologies are critical to achieving the full potential of bipyridine derivatives in the synthesis of advanced functional materials.

## 7. Outlook

Although the synthesis of bipyridine derivatives appears to be simple, research on efficient synthetic methods continues. When using metal catalysts to prepare bipyridine compounds, one strategy is to use bulky ligands, which tend to suppress the coordination of bipyridine and enhance reaction efficiency. Electrochemical synthesis is also being considered, but its applicability is currently limited to specific substrates. Another intriguing approach uses third period nonmetallic elements, which pass through a highly coordinated state. However, a major challenge in this method is the substantial amount of residue generated during the workup. Efforts are underway to address these challenges and further optimize synthetic protocols for bipyridine derivatives.
